# A case of ruptured pancreaticoduodenal artery pseudoaneurysm following endovascular aneurysm repair in a patient with celiac artery stenosis caused by the median arcuate ligament

**DOI:** 10.1016/j.jvscit.2025.101946

**Published:** 2025-08-05

**Authors:** Hironari Shibahara, Jun Yokote, Masato Yamakawa, Masahiro Muto, Ryosuke Fujii, Shinichi Ashida

**Affiliations:** a Department of Cardiovascular Surgery, Ogaki Municipal Hospital, Ogaki, Japan; b Department of Radiology, Ogaki Municipal Hospital, Ogaki, Japan

**Keywords:** Pancreaticoduodenal artery aneurysm, Pseudoaneurysm, Celiac artery stenosis, Median arcuate ligament, Endovascular aneurysm repair

## Abstract

Pancreaticoduodenal artery (PDA) aneurysm is rare. A 79-year-old man with an abdominal aortic aneurysm and celiac artery stenosis caused by median arcuate ligament compression underwent endovascular aneurysm repair. On postoperative day 1, the patient experienced sudden abdominal pain and hypotension. Emergency computed tomography revealed a retroperitoneal hematoma and ruptured pseudoaneurysm at the pancreatic head. Angiography confirmed the rupture of PDA pseudoaneurysm that was treated successfully with coil embolization. Celiac artery stenosis and coverage of inferior mesenteric artery by endovascular aneurysm repair increased dependence on superior mesenteric artery-PDA arcade, resulting in a flow surge that may have precipitated rupture of pseudoaneurysm.

Pancreaticoduodenal artery (PDA) aneurysms and pancreatic artery aneurysms are relatively rare events, accounting for approximately 2% of all splanchnic artery aneurysms.^1^ PDA aneurysm has various causes, including atherosclerosis, infections, pancreatitis, connective tissue disorders, and trauma,^1^, ^2^, ^3^ in addition to stenosis and obstruction of the celiac artery (CA)^3^, ^4^, ^5^, ^6^ caused by median arcuate ligament (MAL) compression.^6^

We present the first reported case of ruptured PDA pseudoaneurysm that occurred after endovascular aneurysm repair (EVAR) for an abdominal aortic aneurysm (AAA) accompanied by CA stenosis caused by the MAL compression.

## Case report

**Fig 1:**
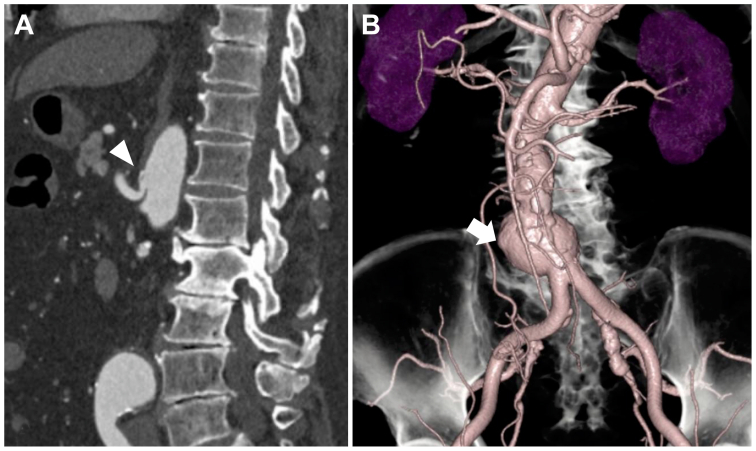
**(A)** Sagittal computed tomography (CT) scan revealing celiac artery (CA) stenosis (*white arrowhead*) caused by the median arcuate ligament. **(B)** Three-dimensional CT scan demonstrating an infrarenal abdominal aortic aneurysm (AAA) with a maximal diameter of 51 mm (*white arrow*).

A 79-year-old man was referred to our institution for an AAA. His medical history included open sigmoidectomy for sigmoid diverticulitis and myocardial infarction. Computed tomography (CT) scan revealed an infrarenal AAA with a maximal diameter of 51 mm, along with CA stenosis caused by the MAL compression (Fig 1, *A* and *B*). No splanchnic artery aneurysms were detected. According to the Japanese Circulation Society guidelines,^7^ invasive treatment is recommended for aneurysms 50 mm or larger (Class IIa). The patient, fully informed of the risks and benefits, desired treatment. Given his age and history of prior laparotomy, EVAR was planned. The procedure was performed through bilateral inguinal puncture using the TREO stent graft (Terumo Aortic). After embolization of the right internal iliac artery with a 14-mm Amplatzer plug, the proximal landing zone was located at the infrarenal abdominal aorta, and the distal landing zone was established at the right external iliac artery. On the left side, the landing zone was situated at the common iliac artery. The stent's main body covered the inferior mesenteric artery (IMA).

**Fig 2:**
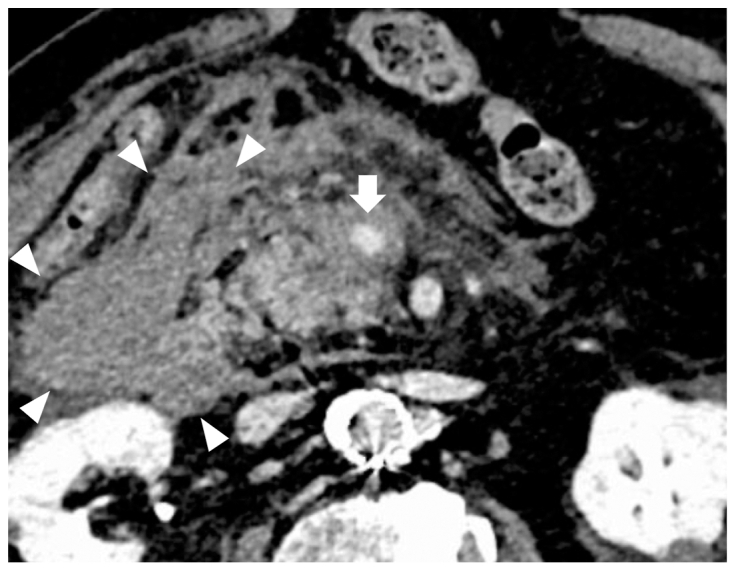
Emergency computed tomography (CT) scan revealing a large hematoma at the retroperitoneum (*white arrowhead*) and a pseudoaneurysm (*white arrow*) on the anterior surface of the pancreatic head.

**Fig 3:**
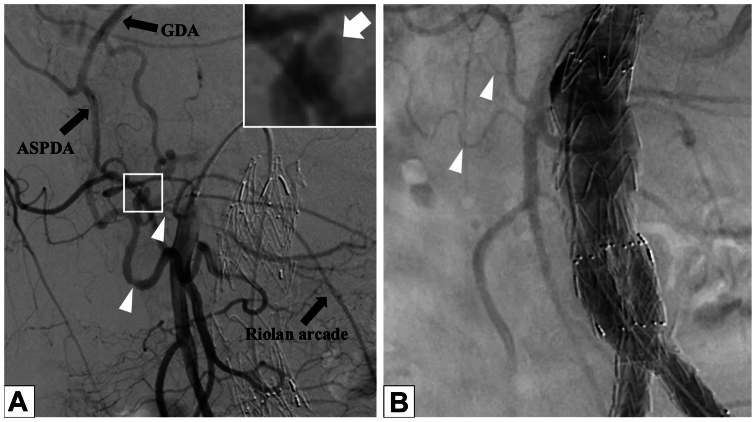
**(A)** Emergency angiography demonstrating a pseudoaneurysm of the anterior superior pancreaticoduodenal artery (*ASPDA*), shown in the inset (*white arrow*), marked dilation of the PD arcade (*white arrowheads*), retrograde flow from the superior mesenteric artery (SMA) to the gastroduodenal artery (*GDA*) and ASPDA via the PD arcade, and dilation of the Riolan arcade. **(B)** Angiography during endovascular aneurysm repair (EVAR) showing a nondilated arcade from the SMA (white arrowhead).

**Fig 4:**
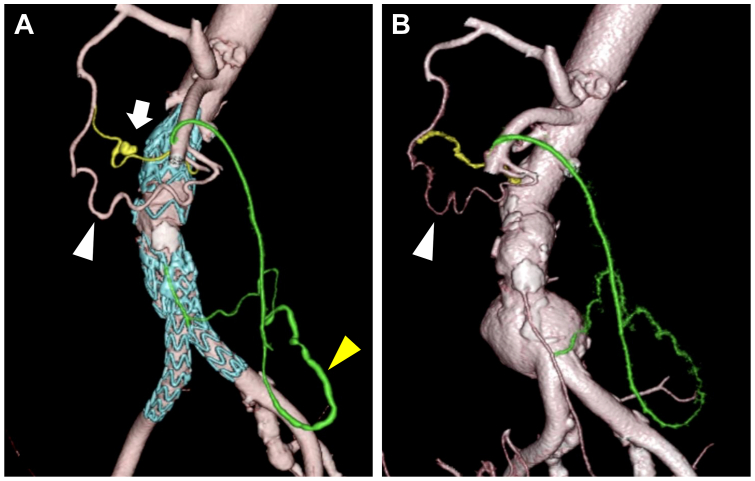
**(A)** Emergency three-dimensional computed tomography (3D-CT) scan showing the vasodilation from the superior mesenteric artery (SMA) to the Riolan arcade (*yellow arrowhead*) and the pancreaticoduodenal (PD) arcade (*white arrowhead*). A pseudoaneurysm of the anterior superior PD artery (PDA) (*white arrow*) was revealed. The vessels in *green* represent the Riolan arcade from the SMA to the inferior mesenteric artery (IMA). **(B)** Preoperative 3D-CT showing that the PD arcade from the SMA (*white arrowhead*) was not dilated, and no obvious PDA aneurysm was detected.

On the operative day, the patient's condition remained stable. However, on postoperative day 1, although the hemoglobin level was maintained at 12.0 g/dL, the patient experienced sudden abdominal pain with a marked decrease in arterial blood pressure to 60 mm Hg by the following early morning. Emergency CT scan revealed a large retroperitoneal hematoma with a pseudoaneurysm on the pancreatic head (Fig 2). Emergency angiography revealed a pseudoaneurysm of the anterior superior PDA with the dilation in the PD arcade (Fig 3, *A*), compared with the previous angiography during EVAR, which showed no dilation of PD arcade (Fig 3, *B*). Urgent embolization of this pseudoaneurysm with coils was performed successfully and achieved good hemostasis. A retrospective comparison of the emergency (Fig 4, *A*) and the preoperative (Fig 4, *B*) CT scans clearly demonstrated the dilation of PD arcade. In addition, the Riolan arcade, which communicates between the superior mesenteric artery (SMA) and IMA, appeared to be more dilated (Fig 4, *A*). Moreover, selective SMA angiography showed the retrograde flow from SMA to the gastroduodenal artery (GDA) and common hepatic artery via the PD arcade (Fig 3, *A*). During emergent angiography, no vasoactive agents or vasodilators were administered.

After treatment, his postoperative course was uneventful, and the patient was discharged on postoperative day 17. The patient provided written consent for the publication of this case report.

## Discussion

The causes of PDA aneurysm vary and include atherosclerosis, infections, congenital defects, pancreatitis, fibromuscular dysplasia, connective tissue disorders, and trauma.^1^, ^2^, ^3^ Occlusion or stenosis of CA has also been reported to cause PDA aneurysms.^3^, ^4^, ^5^, ^6^ Quandalle et al^4^ reported that CA stenosis was associated with PDA aneurysms in 23 of 34 cases. Mano et al^5^ described five consecutive cases of PDA aneurysms associated with CA occlusion. Chivot et al^6^ reported 10 cases of ruptured PDA aneurysms associated with CA stenosis owing to the MAL. In our case, screening for connective tissue disorders was not performed, owing to the patient’s advanced age, unremarkable clinical history, and lack of suggestive findings on preoperative CT scans. All these factors made these diagnoses unlikely.

The mechanism of aneurysm formation is thought to be altered and resulting in increased blood flow in PD arcade. Mano et al described that vortex or helical flow was observed throughout PDA, and oscillatory shear index maps, which reflect more instantaneous wall shear stress vector fluctuation from the main stream direction, showed multiple regions with extremely high oscillatory shear index values distributed heterogeneously throughout the PDA in all patients with PDA aneurysms.^5^ Both the common hepatic artery and GDA had retrograde flow. Flow volume in the GDA and SMA was significantly higher in patients than in control participants. The authors stated that these hemodynamic changes may be associated with PDA aneurysm. Similarly, Chivot et al^6^ reported that selective SMA angiography revealed retrograde blood flow in PDA and GDA in all cases with ruptured PDA aneurysms. Regarding treatment outcomes, they reported that, among 10 patients with ruptured PDA aneurysms, embolization was successful in 8 cases, and 2 patients underwent surgery, with 1 resulting in death. These results suggest that embolization is safe and has a high success rate.^6^

In our case, CA stenosis resulted in compensatory collateralization via the PD arcade. A retrospective comparison of angiographic images from emergency angiography (Fig 3, *A*) and angiography during EVAR (Fig 3, *B*) revealed a marked dilation within the PD arcade on emergency angiography. This vasodilation was interpreted as an indirect indication of increased blood flow from the SMA-PDA arcade. EVAR with coverage of the IMA and embolization of internal iliac artery increased the dependence on the SMA-PDA arcade, resulting in a flow surge that may have precipitated rupture of a PDA pseudoaneurysm.

From an objective standpoint, some understandably concerning aspects of this case may arise. First, regarding the risk of iatrogenic injury: The guidewire was manipulated carefully under continuous fluoroscopy, with the tip always visible. The CA and SMA angulated sharply caudally from the aorta. Given the caudal approach of the guidewire manipulation and this anatomy, inadvertent deep entry into these vessels would be extremely difficult, if not impossible. Second, regarding the unusually rapid aneurysm formation: Chivot et al^6^ reported ruptured PDA aneurysms in 10 patients, with aneurysms detected by CT in 6 cases and by selective SMA angiography in 4 cases. The authors did not specify whether the aneurysms were true or pseudoaneurysms, nor any triggering events, but their rapid presentation with severe symptoms resembles our case. Given the sudden onset after EVAR and the hemodynamic stress in the PD arcade, we diagnosed a pseudoaneurysm that formed rapidly postoperatively. Third, regarding the mild CA stenosis and its relation to increased flow: Previous stated findings^5^^,^^6^ suggest that the formation of the PDA aneurysm may not be due to only the stenosis itself, but rather to the increased hemodynamic stress caused by retrograde flow into PDA. Thus, its cause may be possibly independent of the degree of CA stenosis.

To our best knowledge, there have been no previous reports describing hemodynamic changes associated with CA stenosis owing to MALS in the setting of EVAR. More similar cases could be reported in the future.

## Conclusions

In CA stenosis, EVAR may induce significant hemodynamic changes in the SMA. Therefore, the risk of hemorrhagic rupture through the PD arcade should be carefully monitored.

## Declaration of generative Ai and Ai-assisted technologies in the writing process

During the preparation of this work, the authors used ChatGPT (OpenAI) to improve the readability and language of the manuscript. After using this tool, the authors reviewed and edited the content as needed and take full responsibility for the content of the published article.

## Funding

None.

## Disclosures

None.
